# Bariatric Surgery Patients' Perceptions of Weight-Related Stigma in Healthcare Settings Impair Post-surgery Dietary Adherence

**DOI:** 10.3389/fpsyg.2016.01497

**Published:** 2016-10-10

**Authors:** Danielle M. Raves, Alexandra Brewis, Sarah Trainer, Seung-Yong Han, Amber Wutich

**Affiliations:** ^1^Mayo Clinic/ASU Obesity Solutions, Arizona State UniversityTempe, AZ, USA; ^2^School of Human Evolution and Social Change, Arizona State UniversityTempe, AZ, USA

**Keywords:** weight stigma, dietary adherence, eating behaviors, bariatric surgery, weight loss, obesity, diet, weight bias

## Abstract

**Background:** Weight-related stigma is reported frequently by higher body-weight patients in healthcare settings. Bariatric surgery triggers profound weight loss. This weight loss may therefore alleviate patients' experiences of weight-related stigma within healthcare settings. In non-clinical settings, weight-related stigma is associated with weight-inducing eating patterns. Dietary adherence is a major challenge after bariatric surgery.

**Objectives:** (1) Evaluate the relationship between weight-related stigma and post-surgical dietary adherence; (2) understand if weight loss reduces weight-related stigma, thereby improving post-surgical dietary adherence; and (3) explore provider and patient perspectives on adherence and stigma in healthcare settings.

**Design:** This mixed methods study contrasts survey responses from 300 postoperative bariatric patients with ethnographic data based on interviews with 35 patients and extensive multi-year participant-observation within a clinic setting. The survey measured experiences of weight-related stigma, including from healthcare professionals, on the Interpersonal Sources of Weight Stigma scale and internalized stigma based on the Weight Bias Internalization Scale. Dietary adherence measures included patient self-reports, non-disordered eating patterns reported on the Disordered Eating after Bariatric Surgery scale, and food frequencies. Regression was used to assess the relationships among post-surgical stigma, dietary adherence, and weight loss. Qualitative analyses consisted of thematic analysis.

**Results:** The quantitative data show that internalized stigma and general experiences of weight-related stigma predict worse dietary adherence, even after weight is lost. The qualitative data show patients did not generally recognize this connection, and health professionals explained it as poor patient compliance.

**Conclusion:** Reducing perceptions of weight-related stigma in healthcare settings and weight bias internalization could enhance dietary adherence, regardless of time since patient's weight-loss surgery.

## Introduction

Bariatric surgery is on the rise in the US and globally (Angrisani et al., 2015). The surgery typically triggers massive weight loss, and can immediately and dramatically diminish incidence of morbidities like diabetes (Buchwald and Williams, 2004; Sjöström et al., 2004; Maggard et al., 2005; Dixon et al., 2008; Kalarchian and Marcus, 2015). Bariatric surgery, however, also permanently alters the stomach and intestines and frequently includes long-term deficiencies and absorption issues, which contributes to the view that it is a tactic of “last resort” (Ogden et al., 2005, 2006; Fardouly and Vartanian, 2012; Homer et al., 2016). Moreover, in the years following surgery, research has shown either insufficient weight loss or significant long-term weight regain to be common in some patients, with greater risk of weight regain and obesity-related comorbidities seen after 2 years post-surgery (Sjöström et al., 2004; Magro et al., 2008; da Silva et al., 2016; Peacock et al., 2016). Long-term success at maintaining weight loss after bariatric surgery is multifactorial; however, research has shown that one important contributor is the ability to adhere voluntarily to strict dietary guidelines (Elkins et al., 2005; Kalarchian and Marcus, 2015). These include eating very small, regular portions of food, with an emphasis on lean proteins, fluids, and vegetables; and avoiding high fat/sugar foods (Elkins et al., 2005; Weineland et al., 2012).

A range of research focused on bariatric patients has shown that people find adherence to the dietary recommendations very difficult in the long term (Hsu et al., 1997, 1998; Elkins et al., 2005; Poole et al., 2005; van Hout et al., 2005; Toussi et al., 2009; Snyder et al., 2010; Sarwer et al., 2011; Chesler, 2012; Homer et al., 2016). After the initial 6–12 months post-surgery, as the physical limitations ease somewhat with time, adhering to new, drastically smaller portion sizes is believed to be the primary challenge for most of the men and women who undergo the surgery (MacLean et al., 1983; Miskowiak et al., 1985; Näslund et al., 1988; Andersen and Larsen, 1989; Lindroos et al., 1996; Malone and Alger-Mayer, 2004; Sjöström et al., 2004; Sarwer et al., 2008). Grazing, snacking, binge eating, and emotionally triggered eating are often reported (Kalarchian et al., 2002; Elkins et al., 2005; Poole et al., 2005; Beck et al., 2012; Chesler, 2012; Sheets et al., 2015; Hübner et al., 2016). There are numerous reasons for such behaviors—which end up being labeled “dietary non-compliance”—including psychological, physiological, social, and environmental factors found to have more of an impact with greater time since surgery (Peacock et al., 2016). Lack of support, both before and after surgery, is a major issue, whether that lack is familial, workplace-based, or simply the result of living in an environment in which unhealthy foods and constant social eating are the norm (Benson-Davies et al., 2013).

Disordered eating is typically assessed pre-bariatric surgery by clinicians to determine whether a patient is a good candidate for the surgery, but some patients who have experienced disordered eating prior to surgery are still recommended for surgery, may receive minimal counseling beforehand, and thus may be at risk of disordered eating after surgery. Studies have shown these patients to have poorer post-surgical weight loss and eventual weight re-gain (Hsu et al., 1997, 1998; Elkins et al., 2005; Poole et al., 2005; van Hout et al., 2005; Toussi et al., 2009). Greater general compliance to lifestyle changes has been observed prior to bariatric surgery, supporting the perception that patients are more compliant in the relatively short period prior to surgery than in the long years after (Toussi et al., 2009). Other outside qualitative research suggests that while patients understand dietary changes are essential for long-term success of surgery, they often also have unrealistic expectations of bariatric surgery and hope it will control their eating habits, a stance at odds with clinician expectations that focus on personal responsibility in controlling one's diet (Homer et al., 2016).

The broader dietary literature suggests that weight-related stigma in general can impact dietary decisions and behaviors, precipitating binge eating, skipping meals, early onset-dieting at young ages, “yo-yo” dieting, disordered eating symptoms, emotionally triggered eating, more frequent consumption of convenience foods, and irregular meal times (Puhl and Brownell, 2006; Rosenberger et al., 2007; Puhl and Heuer, 2009; Seacat and Mickelson, 2009; Hübner et al., 2016; Nolan and Eshleman, 2016; Sutin et al., 2016; Vartanian and Porter, 2016). In combination with the psychological and physiological stresses experienced as a result of both chronic weight-related stigma and acute episodes of shame and blame, the process of drawing attention to dietary regulation and ordered eating actually makes these acts far more difficult to adhere to, and may thus ultimately lead to weight (re-) gain (Brewis, 2014; Hunger et al., 2015). Internalized (self-) stigma can also undermine self-esteem and self-efficacy, creating a “why even try?” effect (Corrigan et al., 2009).

Weight-related stigma is a powerful force across diverse societies in the world today, and particularly pernicious in the United States and other Western nations (Braziel and LeBesco, 2001; Campos, 2004; Rogge et al., 2004; Puhl and Heuer, 2009, 2010; Rothblum and Solovay, 2009; Bell et al., 2011; Brewis, 2011, 2014; Farrell, 2011; LeBlasco, 2011; McCullough and Hardin, 2013). Hospital and broader healthcare settings in the United States are often reported by higher body-weight patients to be especially stigmatizing (Mold and Forbes, 2013). Stigma against higher body-weight patients has been documented both in clinician surveys and in patient reports. For example, clinicians have reported they prefer not to treat obese patients (Hebl et al., 2003; Puhl et al., 2009, 2014; Persky and Eccleston, 2011; Phelan et al., 2014, 2015; Tomiyama et al., 2015). Recent surveys show that even obesity specialists themselves can exhibit high implicit and explicit stigma levels (Tomiyama et al., 2015). Surveys with patients who self-identified as obese report they feel unheard and mistreated, and that this discourages them from seeking and following up on services (Rand and Macgregor, 1990; Adams et al., 1993; Olson et al., 1994; Puhl and Brownell, 2006). Weight-related stigma most often has been reported coming from doctors, but it emerges in other healthcare professionals' behavior and beliefs as well (Puhl and Brownell, 2006). The physical clinic environment can be unwelcoming too, as medical equipment, beds, and chairs fail to fit people comfortably (Amy et al., 2006; Puhl and Heuer, 2009; Carels et al., 2010), making them feel they are neither welcome nor “normal” (Brewis et al., 2016).

Bariatric patients, who usually need a clinical designation of “morbid obesity” to qualify for surgery, tend to have long histories of weight-related stigma by the time they decide to begin the pre-surgical preparations (Meana and Ricciardi, 2008; Throsby, 2008; Boero, 2012). Weight-related stigma is far more severe for those individuals who have been categorized (medically and/or socially) as “morbidly obese,” based on the problematic but commonly used Body Mass Index (BMI) scale, which so designates anyone who has a BMI>40 or >35 with comorbidities (Puhl et al., 2007; Carels et al., 2010; Schvey et al., 2011; Durso et al., 2012; Mensinger et al., 2016). This weight-related stigma includes experiences of discrimination within the clinic, and research suggests that weight-related stigma experiences predispose most individuals to *avoid* clinical encounters thereafter (Puhl et al., 2013; Phelan et al., 2015). Opting to undergo bariatric surgery, by contrast, necessitates sustained, intimate interactions with healthcare providers. Therefore, we ask, how do bariatric patients make sense of previous experiences of weight-related stigma vis-à-vis current experiences within the bariatric program? What happens during what could easily be extremely fraught clinical encounters? How might these encounters in turn impact patient success at weight loss maintenance after bariatric surgery?

Prior research suggests that despite feeling stigmatized by healthcare professionals prior to surgery, bariatric patients anticipate improved relationships with their healthcare providers as a *result* of their weight loss (Homer et al., 2016). That same research indicates that bariatric patients tend to be unrealistic and overly hopeful in their perceptions of post-bariatric life, but the point is nonetheless an important one: patients *expect* weight-related stigma to decrease after surgery and its accompanying weight loss. Given the clear links established between experiences of weight-related stigma and compensatory eating, we also ask, when patient weight loss actually results in the expected decrease in weight-related stigma within the clinic setting, is subsequent dietary adherence higher? Do patients who feel more stigmatized even after bariatric surgery report lower dietary adherence?

Combining survey and ethnographic analyses, in this paper, we provide an understanding of the complex relationships between weight stigma and dietary adherence in post-bariatric surgery patients. Our objectives are to evaluate how bariatric patients' perceptions of weight-related stigma impact post-surgical dietary adherence, and understand if weight loss alleviates weight stigma, thereby improving post-surgical dietary adherence. We also explore differences in perception in-patient vs. clinician understandings of weight-related stigma and dietary non-adherence. Notably, almost all qualitative studies of obesity-related stigma have been entirely focused on patients' perspectives, and one goal here was to broaden the view to also assess how clinicians' perspectives might differ, and how the two relate to each other. Since a common recommendation is that changing clinicians' attitudes is key to reducing stigma in healthcare settings (Phelan et al., 2015), this is an important point.

## Materials and methods

This mixed methods study uses quantitative and qualitative data to develop a detailed snapshot of patient and provider experiences within a particular medical system that has bariatric programs located in the American Midwest and Southwest. The quantitative data was drawn from 300 postoperative bariatric surgery patients who went through bariatric surgery in the Midwest or Southwest programs, all of whom completed a detailed survey. The qualitative data relies equally on semi-structured interviews with 35 bariatric patients and on extended participant-observation within the clinical spaces associated with the Southwest location. The qualitative research has been ongoing for over 4 years, and preliminary results from it informed the quantitative assessment.

### Survey (quantitative) data collection

The study sample for the survey consisted of all patients within one single national hospital system with a bariatric program in the Southwest and another in the Midwest. All patients within this system who had undergone any form of bariatric surgery in the prior 5 years (*N* = 994) were sampled. The majority of patients in this system have had the Roux-en-Y gastric bypass; the second most popular surgery is the vertical sleeve gastrectomy. All patients have at least sporadic contact with the healthcare system and clinic, ranging maximally from attending formal on-going support group meetings and regular medical follow-ups to minimally receiving mail-outs several times a year. Before surgery, they all received standardized general dietary recommendations and went through similar pre-surgery informational classes on the fundamentals of nutrition, post-bariatric diet requirements, food labels, emotional eating, food triggers, etc.

For this research, all patients were contacted by mail-out questionnaire, with phone-call follow-ups to encourage participation and assist with completion as needed. The cross-sectional survey was designed specifically for this study. The final sample of returned completed surveys was 298, representing 30% of mail-out. Female and white patients were predominant (as is typical of the bariatric population in this medical system), and patients ranged in age from 23 to 80 (*M* = 52.7, *SD* = 11.9). The Institutional Review Boards of Arizona State University and the relevant hospital both reviewed and approved the human subject protections applied in this study.

### Weight-related stigma measures

#### Reports of weight-related stigma experiences (SSI)

Experiences of felt weight-related stigma within the last 3 months (the reporting window) were assessed by self-report using a 30-item modified version of the 50-item Myers and Rosen Stigmatizing Situations Inventory (SSI) (the full version was too burdensome, based on piloting). The 50-item SSI has been previously validated for use in bariatric populations (Myers and Rosen, 1999), and most recently Vartanian (Vartanian, 2015) validated a brief 10-item version. SSI captures everyday stigmatizing encounters experienced by people with obesity from a variety of sources (e.g., family and friends, strangers, healthcare professionals). This inventory scores each item on a 4-point scale ranging from 0 (“never”) to 3 (“several times”). Cronbach's alpha for the modified 30-item scale was acceptable (α = 0.84).

#### Weight-related stigma in healthcare (HCWS)

To capture weight stigma experiences in healthcare settings specifically, we created a new measure, healthcare weight-related stigma (HCWS), one that collapsed two existing tools—the Interpersonal Sources of Weight Stigma (ISWS) tool, developed by Puhl and Brownell (2006), and the healthcare-specific items on the SSI. The ISWS identifies interpersonal sources of stigma, with patients asked to identify whether they had ever felt stigmatized or discriminated against by their doctors, nurses, dietitians/nutritionists, and mental health professionals (psychologists or social workers) within the previous 3 months. Two items from the SSI included were: “having a doctor make cruel remarks, ridicule you, or call you names” and “having a doctor recommend a diet even if you did not come in to discuss weight loss.” Scores for each item were measured on a 4-point scale ranging from 0 (“never”) to 3 (“several times”). Cronbach's alpha for the modified scale, prior to surgery and after surgery, was acceptable (α = 0.84).

#### Weight bias internalization (WBIS)

Durso and Latner's (2008) Weight Bias Internalization Scale (WBIS) was used to capture internalized weight-related bias (“self-stigma”). The original 11-item WBIS, evaluating the degree to which an individual believes that negative societal prejudice and attitudes apply to them personally, was shortened to reduce respondent burden to 8-items assessed on a 4-point Likert scale from “strongly disagree” to “strongly agree.” Cronbach's alpha for the modified scale was acceptable (α = 0.78). The scale asks patients to rate self-stigma for the last 3 month period.

### Dietary adherence measures

Dietary adherence was assessed three ways. We relied on patient self-reports of how well they were following clinician guidelines, their dietary intake as reported by them on a food frequency questionnaire, and their eating behaviors as reported on a disordered eating scale.

#### Patient dietary success—self-assessed (PSA)

In order to assess patient dietary success as self-assessed (PSA) we asked each respondent how well he or she had followed the dietary recommendations provided to him or her after surgery by their bariatric dietitians over the preceding 3 months. Responses were on a 10-point Likert scale ranging from 1 (not at all) to 10 (all the time). Average scores from the current study (6.2 ± 2.3), obtained on average at 20.7 months post-surgery, were comparable to those reported in 200 postoperative bariatric surgery patients at 20 weeks post-surgery (6.5 ± 2.0; valid range 1–9), using a similar question, “How well are you following the diet plan given to you by the dietitian?” (Sarwer et al., 2008).

#### Dietary recommendation adherence (FFQ-DA)

The Food Frequency Questionnaire (FFQ) is a standard tool for capturing frequency and quantity of dietary intake in survey format. This version was adapted from the nutritional pyramid for Roux-en-Y gastric bypass patients and estimates the frequency of consumption within the last month of typical food and beverage items (Moizé et al., 2010). For this analysis, we computed the frequency of consumption of five food and beverage items identified within this bariatric clinic program as items to avoid consuming any time after surgery. In the FFQ, each of these five dietary items was estimated on a 9-point frequency scale from 0 (consumed less than once per month) to 8 (consumed six or more times per day). These items were: refined grains, salty snacks, sweets, sugar-sweetened beverages, and alcoholic beverages. For clarity, the measure of dietary recommendation adherence used in our analysis, hereafter termed FFQ-DA, was summed inversely to the scale on the FFQ so that a higher value represents better adherence to recommendations. Therefore, a higher FFQ-DA score shows greater adherence to dietary recommendations (and lower intake of foods to avoid) and a lower value indicates worse adherence to dietary recommendations (and greater intake of foods to avoid). The summary scores range from 0 (highly frequent consumption of all) to 40 (no consumption).

#### Non-disordered eating behaviors (DEBS)

The Disordered Eating after Bariatric Surgery (DEBS) tool is a validated 7-item, self-reported screener for disordered eating behaviors post-bariatric surgery (Weineland et al., 2012). Eating behaviors were assessed with respect to the previous 3 months. After piloting, we adjusted the scale wording slightly to improve patient understanding and ease. A higher value indicates greater disordered eating behaviors and vice versa. Cronbach's alpha for the modified scale was acceptable (α = 0.78).

### Weight variables

The main weight variable of interest was weight lost, as a percentage of pre-surgical body mass index (BMI). Height (in feet and inches) and weight (in pounds) were based on self-report. Bariatric patients are reported to be relatively accurate in reporting body weight compared to general populations because they must track themselves so carefully (Christian et al., 2013). BMI (kg/m^2^) was calculated from height and weight. Percent change in BMI (from prior to surgery to the time the survey was administered) was standardized such that a 10% change in BMI corresponded to a one-unit change in the variable.

### Covariates

College education was expressed as a dichotomous variable such that 0 equals less than college completion and 1 equals college completion. Higher income was expressed as a dichotomous variable such that 0 equals less than $100,000 annual household income and 1 equals $100,000 annual household income or greater. Other key covariates are listed in Table 1.

**Table 1:** **Descriptive statistics of outcomes, predictors, and covariates**.

**Variable**	**N^a^**	**Mean**	***S. D*.**	**Min**	**Max**
Self-assessed diet plan adherence (PAS)	292	6.22	2.31	1	10
Diet plan adherence (FFQ-DA)	237	31.16	4.99	15	40
Disordered eating (DEBS)	279	8.65	6.07	0	36
Internalized stigma (WBIS)	283	30.34	5.87	15	45
Healthcare stigma (HCWS)	275	0.79	2.10	0	17
Experienced stigma (SSI)	218	8.28	7.12	4	70
Gender (Male)	289	0.23	0.42	0	1
Difference in BMI (%)	290	−3.36	0.98	−6.79	−0.17
Current BMI (kg/m^2^)	291	30.54	6.54	16.04	70.37
Current age (years)	288	53.79	12.76	19	82
Time since surgery (months)	293	20.76	12.29	0	68
College^b^	295	0.76	0.43	0	1
High income^c^	289	0.43	0.50	0	1

a *Number of cases with valid values in each variable out of total 300 cases*.

b *College is a dichotomous variable such that 0, < college completion and 1, >college completion*.

c *High income is a dichotomous variable such that 0, < $100,000 annual household income and 1, > $100,000 annual household income*.

### Survey (quantitative) analysis

Survey data for a total of 300 post-bariatric surgery patients was analyzed using SAS 9.4. Multiple linear regression was employed to assess the relationships between weight-related stigma measures and the three dietary adherence measures, and also how weight loss (BMI percent change) influenced the stigma variables. Variables controlled include gender, current age, time since surgery, education status, income status, current body size (BMI), and percent change in BMI. SSI prior to surgery, and experiences of weight-related stigma in healthcare settings prior to surgery. There were no missing values for variables in each of the respective models. Confidence intervals were set to 95% and a *p* ≤ 0.05 was considered statistically significant.

### Ethnographic (qualitative) data collection

In addition to the survey data, this study draws on two qualitative datasets: semi-structured interviews and ethnographic field notes. The qualitative phases of this research were conducted with the current patient population at the Southwest clinic site within the medical system we surveyed. Patients were recruited into the study after enrolling in the pre-surgical preparatory program prior to bariatric surgery or in the 24 months post-surgery. The interview sample (*N* = 35) was reflective of the overall bariatric clinic population in terms of gender, ethnicity, and age. Of the interviewees, 33 (eight men and 26 women) had a Roux-en-Y gastric bypass and two interviewees (both women) opted for a vertical sleeve gastrectomy. The interview data set consisted of 35 first interviews, follow-up interviews with 27 of the original participants 4–8 months after the first interviews, and an additional follow-up interview with 25 of the original participants 4–8 months after the second interviews. All interviews were conducted by a trained and experienced ethnographer (ST), and systematically covered a series of discussion domains that included food/eating and experiences of weight-related stigma. Interviews took 45–120 min, and were audiotaped and then fully transcribed using standard protocols (McLellan et al., 2003). In addition to interviews, extensive participant-observation was conducted over multiple years in the same clinic bariatric practice from which interviewees were recruited. Participant observation included attending the required pre-operative nutrition classes and post-surgery bariatric support group meetings, as well as informal conversations with medical providers and staff. Field notes were taken during or immediately after all participant observation activities and these texts were included in the qualitative dataset. Triangulation between views expressed by patients in individual recorded interviews vs. in public forums like the support group meetings, as well as those expressed by providers in public forums like the support group meetings vs. private informal conversations were key in moving data collection and analysis forward. Formal, audio-recorded interviews with providers were not allowed as part of the data collection, but the informal conversations yielded comparable themes.

### Ethnographic (qualitative) analysis

Data consisted of interview transcripts and field notes, and analyses subsequently relied on structured coding procedures and thematic characterizations of the coded segments (Krippendorff, 2012; Benard et al., 2016), using MAXQDA software. Relevant codes were identified from the literature and a structured codebook was developed following the procedures outlined by MacQueen et al. (1998). The codebook included detailed definitions, typical exemplars, atypical exemplars, and marginal/irrelevant examples from the texts to illustrate the range of meanings assigned to themes. We assessed inter-rater reliability of codes using a random sample of 40 segments from our preliminary interviews, and final code definitions reached a high level of inter-rater agreement (kappa > 0.7). Core analytic tools included thematic comparison (Boeije, 2002; Benard et al., 2016). Codes in the current analyses include (1) weight stigmatizing experiences within a healthcare setting and (2) patient dietary adherence before and after surgery. We summarized the range of themes present in the interview and ethnographic data and employed typical exemplar segments that demonstrate key dimensions of these themes.

## Results

### Quantitative results

The descriptive statistics of the outcomes, predictors, and covariates of the study patients are shown in Table 1.

#### Dietary adherence after bariatric surgery

The mean PSA score was 6.2 ± 2.3, which indicates that on average respondents believed that they were moderately successful at following the dietary recommendations made to them by their bariatric dietitians. Similarly, the FFQ-DA score indicates that patients reported adhering moderately well to avoiding “forbidden foods/beverages” listed by their dietitians as foods not to consume after surgery (*M* = 31.2 ± 5.0). The average DEBS score shows that post-bariatric surgery patients do not exhibit high disordered eating habits after surgery (*M* = 8.7 ± 6.1); however, 16% of 279 cases exhibited high disordered eating habits based on the criteria of 1 *SD* above the mean DEBS score.

Bivariate correlations show that all dietary adherence variables have moderate significant associations (see Table 2). PAS and FFQ-DA were positively and significantly associated, suggesting that patients that reported successfully following dietary recommendations made to them by their dietitians also reported avoiding “forbidden foods/beverages” listed by their dietitians. Significant inverse relationships were observed between DEBS and both PAS and FFQ-DA, indicating that patients that exhibited higher disordered eating habits did not report adhering well to dietary recommendations made to them by their dietitians and consumed more “forbidden foods/beverages” listed by their dietitians.

**Table 2:** **Correlations between diet plan, disordered eating, and weight-stigma indices**.

**Variables**	**1**	**2**	**3**	**4**	**5**	**6**	**7**
1. Self-assessed diet plan adherence (PAS)	1.00						
2. Diet plan adherence (FFQ-DA)	0.27^**^	1.00					
3. Disordered eating (DEBS)	−0.46^**^	−0.42^**^	1.00				
4. Internalized stigma (WBIS)	−0.19^*^	−0.18^*^	0.35^**^	1.00			
5. Healthcare stigma (HCWS)	−0.03	−0.02	0.12	0.11	1.00		
6. Experienced stigma (SSI)	−0.08	−0.02	0.16^*^	0.20^*^	0.50^**^	1.00	
7. Weight lost (BMI %)	−0.13	0.08	0.06	−0.04	−0.08	0.01	1.00

*Total number of cases used for the correlation analysis is 167; ^†^p < 0.10*;

* *p < 0.05*;

** *p < 0.01; two tailed*.

On average, patients completed the survey within 2 years after surgery; therefore it is possible that these eating behaviors could change as they move further away from the time of their surgery. For example, multiple linear regression analyses showed that there were significant effects of time since surgery on all three dietary adherence measures, PAS (coef. = −0.04 [−0.06; −0.01], FFQ-DA (coef. = −0.13 [−0.20; −0.07]), and the DEBS (coef. = 0.13 [0.06; 0.19]), indicating that patients with greater time since surgery believed themselves to be less successful at following dietary recommendations made to them by their dietitians, reported less adherence to “forbidden foods/beverages” listed by their dietitians as foods to avoid, and exhibited greater disordered eating habits (see Tables 3–5). Gender was also a significant contributor to PAS (coef. = −0.83 [−1.59; −0.07]) and FFQ-DA (coef. = −2.79 [−4.61; −0.96]), with males reporting worse self-assessed dietary adherence and consuming more foods and beverages they had been advised to avoid (see Tables 3, 4). High income showed a positive significant association with DEBS scores after controlling for covariates (see Table 5).

**Table 3:** **Estimated effects of weight stigma indices on self-assessed diet plan adherence (PAS)**.

	**Model 1**		**Model 2**		**Model 3**		**Model 4**	
	**B**	**95% C.I**.	**B**	**95% C.I**.	**B**	**95% C.I**.	**B**	**95% C.I**.
**WEIGHT STIGMA**								
SSI	−0.04	[−0.10; 0.02]					−0.02	[−0.09; 0.04]
HCWS			−0.09	[−0.29; 0.12]			−0.03	[−0.26; 0.20]
WBIS					−**0.07**	[−0.13; −0.01]	−**0.07**	[−0.12; −0.01]
**COVARIATES**								
College education	−0.13	[−0.90; 0.65]	−0.09	[−0.86; 0.69]	−0.02	[−0.79; 0.75]	−0.04	[−0.81; 0.73]
High income	0.04	[−0.64; 0.72]	0.11	[−0.56; 0.78]	0.03	[−0.63; 0.69]	−0.02	[−0.69; 0.66]
Time since surgery	−**0.04**	[−0.07; −0.02]	−**0.04**	[−0.07; −0.02]	−**0.04**	[−0.06; −0.01]	−**0.04**	[−0.06; −0.01]
Gender (Male)	−**0.82**	[−1.59; −0.05]	−**0.78**	[−1.55; −0.01]	−**0.81**	[−1.57; −0.05]	−**0.83**	[−1.59; −0.07]
Current age	−0.01	[−0.03; 0.02]	0.00	[−0.03; 0.02]	−0.01	[−0.04; 0.02]	−0.01	[−0.04; 0.02]
Current BMI	0.00	[−0.05; 0.06]	0.00	[−0.06; 0.05]	0.00	[−0.06; 0.05]	0.00	[−0.05; 0.06]
Intercept	**8.03**	[5.58; 10.47]	**7.79**	[5.38; 10.21]	**10.03**	[7.03; 13.03]	**10.07**	[7.06; 13.09]
*F*-value	2.29^*^		2.14^*^		2.96^**^		2.39^*^	
*N*	187		187		187		187	

*Bold if significant at the 0.05 level of significance; ^†^p < 0.10*;

* *p < 0.05*;

** *p < 0.01*;

*two tailed; C.I., Confidence Interval*.

**Table 4:** **Estimated effects of weight stigma indices on diet plan adherence (FFQ-DA)**.

	**Model 1**		**Model 2**		**Model 3**		**Model 4**	
	**B**	**95% C.I**.	**B**	**95% C.I**.	**B**	**95% C.I**.	**B**	**95% C.I**.
**WEIGHT STIGMA**								
SSI	−0.05	[−0.19; 0.08]					−0.04	[−0.20; 0.11]
HCWS			−0.04	[−0.51; 0.43]			0.08	[−0.46; 0.62]
WBIS					−0.13	[−0.26; 0.01]	−0.12	[−0.26; 0.02]
**COVARIATES**								
College education	−1.80	[−3.70; 0.09]	−1.76	[−3.66; 0.13]	−1.56	[−3.45; 0.33]	−1.60	[−3.50; 0.31]
High income	−1.36	[−2.96; 0.25]	−1.25	[−2.84; 0.34]	−1.39	[−2.97; 0.19]	−1.47	[−3.07; 0.14]
Time since surgery	−**0.14**	[−0.20; −0.08]	−**0.14**	[−0.20; −0.08]	−**0.13**	[−0.19; −0.07]	−**0.13**	[−0.20; −0.07]
Gender (Male)	−**2.71**	[−4.54; −0.88]	−**2.67**	[−4.49; −0.84]	−**2.76**	[−4.57; −0.95]	−**2.79**	[−4.61; −0.96]
Current age	0.06	[−0.00; 0.13]	**0.07**	[0.00; 0.14]	0.06	[−0.01; 0.13]	0.06	[−0.01; 0.13]
Current BMI	0.04	[−0.10; 0.19]	0.03	[−0.11; 0.17]	0.04	[−0.10; 0.17]	0.05	[−0.10; 0.19]
Intercept	**32.45**	[26.43; 38.47]	**32.12**	[26.14; 38.09]	**35.85**	[28.71; 43.00]	**35.92**	[28.73; 43.10]
*F*-value	4.89^**^		4.79^**^		5.38^**^		4.17^**^	
*N*	162		162		162		162	

Bold if significant at the 0.05 level of significance; ^†^p < 0.10; ^*^p < 0.05;

** *p < 0.01*;

*two tailed; C.I., Confidence Interval*.

**Table 5:** **Estimated effects of weight stigma indices on disordered eating (DEBS)**.

	**Model 1**		**Model 2**		**Model 3**		**Model 4**	
	**B**	**95% C.I**.	**B**	**95% C.I**.	**B**	**95% C.I**.	**B**	**95% C.I**.
**WEIGHT STIGMA**								
SSI	**0.25**	[0.09; 0.42]					**0.21**	[0.03; 0.39]
HCWS			0.40	[−0.17; 0.98]			−0.02	[−0.64; 0.61]
WBIS					**0.31**	[0.16; 0.46]	**0.28**	[0.13; 0.43]
**COVARIATES**								
College education	−0.77	[−2.90; 1.36]	−0.90	[−3.08; 1.27]	−1.27	[−3.37; 0.83]	−1.16	[−3.23; 0.92]
High income	1.59	[−0.26; 3.44]	1.11	[−0.75; 2.97]	1.42	[−0.38; 3.22]	**1.81**	[0.01; 3.61]
Time since surgery	**0.14**	[0.07; 0.21]	**0.14**	[0.07; 0.21]	**0.12**	[0.05; 0.19]	**0.13**	[0.06; 0.19]
Gender (Male)	0.60	[−1.50; 2.71]	0.39	[−1.76; 2.54]	0.58	[−1.49; 2.65]	0.75	[−1.30; 2.79]
Current age	−0.03	[−0.11; 0.04]	−0.05	[−0.13; 0.02]	−0.03	[−0.10; 0.05]	−0.01	[−0.08; 0.07]
Current BMI	−0.04	[−0.20; 0.12]	0.02	[−0.14; 0.18]	0.02	[−0.14; 0.17]	−0.04	[−0.19; 0.12]
Intercept	6.13	[−0.53; 12.80]	**7.74**	[1.03; 14.44]	−2.28	[−10.51; 5.95]	−2.83	[−10.95; 5.30]
*F*-value	4.04^**^		2.83^**^		5.02^**^		4.77^**^	
*N*	181		181		181		181	

Bold if significant at the 0.05 level of significance; ^†^p < 0.10; ^*^p < 0.05;

** *p < 0.01*;

*two tailed; C.I., Confidence Interval*.

#### Weight-related stigma and dietary adherence

Bivariate correlations show that WBIS scores were significantly associated with all three measures of dietary adherence (see Table 2). WBIS scores showed a significant inverse association with PAS (coef. = −0.19, *p* ≤ 0.05) and FFQ-DA (coef. = −0.18, *p* ≤ 0.05) indicating that patients that reported greater weight bias internalization believed themselves to be less successful at following dietary recommendations made to them by their dietitians and reported lower adherence with respect to “forbidden foods/beverages” listed by their dietitians as foods to avoid. WBIS scores showed a moderate significant and positive association with DEBS (coef. 0.35, *p* ≤ 0.01) indicating that patients that reported greater weight bias internalization exhibited greater disordered eating habits.

Overall, results from multiple linear regression models show that there were significant effects of weight-related stigma on dietary adherence. WBIS scores were significantly associated with PAS scores (coef. = −0.07 [−0.12; −0.01] (see Table 3) and DEBS scores (coef. = 0.28 [0.13; 0.43]) (see Table 5) after controlling for all other weight-related stigma variables and potential confounders. Therefore, patients with higher WBIS reported worse self-assessed dietary adherence and exhibited greater disordered eating behaviors. Similarly, SSI scores were significantly and positively associated with DEBS scores (coef. = 0.21 [0.03; 0.39]) after controlling for all other weight-related stigma variables and potential confounders (see Table 5). Therefore, patients with greater overall experiences of everyday, generalized weight-related stigma exhibited greater disordered eating behaviors.

HCWS scores, however, were not significantly associated with any measure of dietary adherence. This finding suggests that feelings and experiences of weight-related stigma from healthcare professionals did not impact patients' ability to follow overall dietary recommendations after bariatric surgery.

#### Weight loss and weight-related stigma

Bivariate correlations show that BMI percent change was not significantly associated with any measure of weight-related stigma (see Table 2). Similarly in multiple linear regression models, BMI percent change after bariatric surgery had no effect on any of the three measures of weight stigma (see Table 6). While BMI percent change was not significantly associated with weight stigma after surgery, current BMI was significantly and positively associated with SSI score (coef. = 0.57 [0.41; 0.73]) and HCWS score (coef. = 0.13 [0.09; 0.17] suggesting that patients with a higher BMI experienced more general and healthcare weight-related stigma. Current age was a significant contributor to WBIS score (coef. = −0.10 [−0.15; −0.04]), such that younger patients exhibited more weight bias internalization.

**Table 6:** **Multiple linear regression estimates of the effects of BMI on SSI, HCWS, and WBIS among post-bariatric surgery patients**.

	**On SSI, now**		**On HCWS, now**		**On WBIS**	
	**Est**.	**95% C.I**.	**Est**.	**95% C.I**.	**Est**.	**95% C.I**.
Difference in BMI	−0.77	[−1.78; 0.25]	−0.29	[−0.58; 0.00]	−0.52	[−1.36; 0.32]
Current BMI	**0.57**	[0.41; 0.73]	**0.13**	[0.09; 0.17]	−0.01	[−0.14; 0.13]
Gender (Male)	−1.45	[−3.60; 0.70]	−0.19	[−0.78; 0.40]	−0.70	[−2.38; 0.99]
Current age	−0.07	[−0.14; 0.01]	0.01	[−0.01; 0.03]	−**0.10**	[−0.15; −0.04]
College education	−0.06	[−2.27; 2.15]	0.21	[−0.40; 0.82]	0.64	[−1.10; 2.38]
High income	−**2.23**	[−4.09; −0.38]	−0.17	[−0.69; 0.34]	−1.19	[−2.69; 0.31]
Time since surgery	−0.06	[−0.13; 0.02]	−**0.02**	[−0.05; 0.00]	0.05	[−0.01; 0.10]
Intercept	−5.40	[−14.33; 3.54]	−**4.05**	[−6.51; −1.59]	**33.07**	[25.66; 40.49]
*F*-Value	10.36^**^		6.04^**^		2.71^*^	
*N*	200		252		258	

*Bold if significant at the 0.05 level of significance; ^†^p < 0.10*;

* *p < 0.05*;

** *p < 0.01*;

*two tailed; C.I., Confidence Interval; SSI, Stigmatizing Situations Inventory; HCWS, Healthcare Weight–Related Stigma; WBIS, Weight Bias Internalization Scale*.

### Qualitative results

Two ethnographic datasets resulted from this project: audio recordings of 87 interviews with 35 participants and field notes from multiple years of participant observation within the clinic. Both types of data were vital to the identification of powerful themes that ran through patient and provider experiences.

#### Dietary adherence after bariatric surgery

We have analyzed our qualitative data on participant eating habits in detail elsewhere (Trainer et al., under review). Here, we highlight key themes that emerged and aid in the interpretation of the quantitative data. First, most people in the pre-surgery required behavioral change classes, as well as in the bariatric support group meetings, focused on the ways in which they adhered to the diet mandated by the clinic both pre- and post-surgery, but one-on-one anonymized conversations with people at different points along the surgical trajectory revealed a great deal of deviation from the suggested standards. The deviations not only included high calorie “slider foods” (ice cream, peanut butter, alcohol, etc.,) and over-consumption, but also under-consumption, meal skipping, omission of vitamin supplements, and under consumption of water. The qualitative data also clearly indicated, however, that people were aware of their deviations and were usually concerned about them—the concern simply did not always translate into healthy behaviors, particularly when the men and women were coping with hectic work schedules, family demands, travel, and social settings that made rigid adherence to the diet difficult (e.g., work banquets).

#### Participant experiences of weight-related stigma

Weight-related stigma from healthcare professionals registered as important in both the semi-structured interviews and the more general discussions that occurred in the behavioral change classes, support group meetings, and conversations with healthcare providers. Importantly, none of the reported instances of weight stigma were specific to the bariatric providers, but instead stemmed primarily from past and/or current experiences with EMTs and ER doctors, OB-GYN doctors and nurses, general practitioners (GP), and (non-bariatric) surgeons.

The most commonly reported type of weight stigma from a healthcare professional is typified by Amy's account. In response to an interview prompt, Amy said that about 10 years previously, she went to see her GP for neck problems. *I thought maybe I was having some kind of neck problem because I worked in a factory. And so I said, “Well, what is that?” And he said, and this was in Tennessee and I'm telling you I got really pissed off, he said, “That's fat. You just love the pork, don't you?”* Then, Amy reported, he slapped her on the area of her neck he had identified as her “fat pack.” Understandably, Amy looked for another GP. *And so I found me another doctor…and that's the one who of course…said if you were dating, you would probably be more concerned [about your weight]…They were white little country doctors. That sounds racist, but they were now that I think about it, you know?*

Amy, who was both female and African American, had no similar complaints to make about her healthcare in the years since those incidents. She had, however, avoided this particular physician demographic.

Other people, in interviews, support group meetings, and pre-surgical classes, reported being chastised (pre-bariatric surgery) for their weight after accidents that required emergency personnel to lift them into an emergency vehicle and/or transfer them to a hospital. Another common complaint was that an individual would attempt to be treated (pre-bariatric surgery)—in an ER or by their regular GP—for an illness unrelated to their weight and would receive a lecture about his or her weight from that healthcare provider. As one woman remarked, *You know, you go in there, “I got a headache.” “It's because you're fat.” “My toes hurt.” “It's because you're fat.”* That same woman, however, was extremely complimentary about the bariatric program providers—and indeed, the entire medical network. *One thing I really like about [it] is the customer service. And you don't feel like you're on the clock*, she said. Others echoed her sentiments.

Often, people reported that, before their enrollment in the bariatric program, their healthcare providers were reluctant to bring up the subject of weight but felt duty-bound to do so. Dallas, for example, responded to an interview prompt by saying, *Oh, I've had doctors tell me, “You're fat. Lose the weight.” But they're also personal friends of mine. “You're getting fatter and you need to get that weight off. You need to get out and exercise.” He [Dallas' favorite primary care provider] says, “I know I'm talking to a wall right now.”* Many participants mentioned that providers would say something rote, along the lines of, “you're technically obese and you need to lose weight in order to be healthy,” then, swiftly move on to other subjects. In other words, the subject was clearly something that many healthcare professionals felt uncomfortable and ill-equipped to handle.

Clinic-based weight stigma, as opposed to other sources of stigma (such as from family members), appeared less frequently in the interview narratives and fieldwork notes about pre-surgery life as a person with obesity. Moreover, because it appeared so infrequently—and most of the stories of encountering doctor- or nurse-bias were not based on recent events—there was very little difference in people's reports of their experiences with healthcare professionals *after* surgery.

#### Stigma, support, and adherence after bariatric surgery

Participants in the interviews did express concerns about their interactions with dieticians and mental health professionals encountered within the bariatric program. None of the issues, however, centered on weight-related stigma, but rather on differences in patient vs. provider notions of dietary and exercise adherence. From the provider perspective, the “rules” of engagement were simple: in opting for a life- and body-altering surgery, patients should also be making a lifetime commitment to identify and change emotional food triggers, engage in mindful eating, follow the rules of soft foods and tiny portions for the first 6 months post-surgery, and then cap their eating thereafter at 1200 calories a day, while avoiding all high-fat, high-sugar “slider foods.” From the patient perspective, adherence was not that simple: social, work, and familial demands sometimes made adherence difficult and emotional/mindless eating sometimes prevailed. These differences in perspective produced tension and we observed that this tension sometimes increased non-adherence on the part of some patients. Interestingly, some of our richest data in this area emerged from triangulating data from patients who self-reported as extremely adherent, but who would then comment on other patients perceived to be less adherent, with our own observations of both sets of patients.

Moreover, felt stigma itself could shift after surgery. People reported in support group meetings and in interviews, for example, that “regular” (i.e., non-bariatric) healthcare professionals did not always handle the particular needs produced by their past history of obesity and current status as a bariatric patient. For instance, many women in particular reported that they wished they could continue seeing a mental health professional after bariatric surgery, in order to better assess the profound changes they were experiencing, but that most psychologists and psychiatrists they saw were unfamiliar with bariatric surgery and ill-equipped to handle their cases. Indeed, after one support group meeting, one woman gave the name of her (out-of-network) psychologist, who specialized in bariatric patients, to a number of other women who had expressed dissatisfaction with their previous experiences. Thus, we see that stigma among healthcare providers is more nuanced than straightforward weight-related stigma, because negative attitudes toward bariatric surgery also enter the equation.

## Discussion

At the outset of this paper, we asked a number of guiding questions: What is the relationship between weight-related stigma and post-surgical dietary adherence? Does weight loss reduce weight-related stigma, thereby improving dietary adherence? How do healthcare providers and patients perceive adherence and weight-related stigma in healthcare settings?

The analyses suggest that post-bariatric patients perceive weight-related stigma as coming from many sources, including from their healthcare professionals. The percentage of patients in the current study who reported in the survey ever feeling stigmatized by doctors (62%) and nurses (45%) is comparable to prior research (Puhl and Brownell, 2006). Having observed this specific clinical practice at length, we suggest this finding is due to these patients having more intense ongoing contact with such professionals. This may include lingering differences of opinion concerning dietary adherence, rather than actively more stigmatizing treatment. Moreover, the qualitative data indicates that out-of-network mental health professionals do not always engage with the specific needs of bariatric patients, from the patient perspective.

More broadly, the findings confirm that clinic settings in general are perceived by some patients who are (or have been in the recent past) higher body weight, as stigmatizing. The issue of how to address this is complicated by the fact that their healthcare providers see it as their medical duty to raise the issue of weight but do not always do so in ways that are perceived to be sensitive or relevant to patients.

With respect to dietary adherence, our survey results reveal that weight bias internalization and reports of stigmatizing experiences of weight-related stigma are correlated with patients' ability to adhere to their post-surgical dietary recommendations. However, reports of stigmatizing feelings and experiences of weight-related stigma within healthcare settings did not. This makes sense in the context of a group of patients who have undergone significant weight loss: they would be experiencing less weight-related stigma in their interactions in healthcare settings if those are at least in part determined by the target's body weight. Results from this study are congruent with other research that shows that younger patients report greater weight bias internalization (Durso and Latner, 2008). Other variables that were correlated with dietary adherence included time since surgery and gender. Patients with greater time since surgery reported worse dietary adherence on all measures compared to those with less time since surgery. The broader literature confirms that greater time since surgery is a contributor to dietary non-compliance (Hsu et al., 1997, 1998; Elkins et al., 2005; Poole et al., 2005; van Hout et al., 2005; Toussi et al., 2009; Snyder et al., 2010; Sarwer et al., 2011; Chesler, 2012; Homer et al., 2016) and an independent risk factor for postoperative weight re-gain (da Silva et al., 2016). Our results show that men reported adhering less to recommendations made to them by their dietitians and reported less avoidance of “forbidden foods/drinks.” Our qualitative data reveals that patients must juggle many demands on their time on a daily basis and sometimes make active decisions to sacrifice dietary adherence, although the gendered difference needs to be explored further.

Results from our survey echo prior research (Mustillo et al., 2012) in showing that feelings of stigma linger long after weight loss, and even after frequent exposure to stigmatizing events. There are several possible reasons for this. One, even after drastic weight loss, post-bariatric surgery patients on average are still usually clinically “obese” (body mass index ≥30), which may continue to influence the ways in which professionals react to and interact with them. Two, research also shows that bariatric surgery is often characterized as a “lazy,” “low-effort” weight loss method by mainstream U.S. society, including by some healthcare professionals (Fardouly and Vartanian, 2012), which may negatively influence clinic encounters after surgery. Three, memories of stigmatizing encounters linger for participants, even after substantial weight has been lost, and this may color the ways in which they perceive their current encounters. Lastly, debates over dietary adherence within the clinical encounter may be perceived as stigmatizing by patients – but not by providers.

## Strengths and limitations

This study has key limitations, including that all quantitative data was collected via self-report. The tools used in the current study to measure weight stigma and disordered eating on the survey were either modified or adapted from validated tools, thereby limiting the ability for true comparability with other studies. Also, the present survey analyses were limited to cross-sectional associations; prospective studies are required to more robustly evaluate these relationships. While traditional methods of dietary assessment rely on self-report, the limitations associated with these methods are well-known, particularly under-reporting of actual consumption (Johnson, 2002). This limitation seems to extend similarly to populations defined by their higher weight status (Lichtman et al., 1992; Heitmann and Lissner, 1995; Mendez et al., 2011) and to bariatric patients (Silver et al., 2006). This study has several strengths, including the use of mixed methods. The effort to characterize the patients in the sample in such a way as to be able to control for several confounders in the quantitative analyses is an advantage and the use of ethnographic data provides a more nuanced understanding of some of the stigma measures. This was also the first study to assess the relationships between multiple measures of weight-related stigma and dietary adherence in a large sample of post-bariatric surgery patients in a multicenter registry, thereby increasing our understanding of many interlinking behaviors and psychosocial measures experienced by this population.

## Conclusion

The current study provides quantitative and qualitative evidence of post-surgical bariatric patients dealing with persistent weight stigma despite massive weight loss, and demonstrates that internalized weight-related stigma and reports of general stigmatizing experiences of weight-related stigma negatively impact eating behaviors across many years following surgery.

The survey results suggest the solution to persistent weight-related stigma is not solely related to changing immediate clinician and other healthcare professional attitudes and behaviors during and after bariatric surgery. In fact, recent stigmatizing experiences of weight-related stigma in healthcare settings did not significantly impact dietary adherence. Rather, the forms of stigma that matter for dietary adherence—internalized stigma and experiences of generalized weight-related stigma—are built over time through multiple feelings of being mistreated and rejected in both healthcare and non-healthcare settings. Reducing weight-related stigma of healthcare providers in all the healthcare settings that people with high body weight encounter must, of course, be a priority. Providing more resources to combat generalized feelings of stigma in pre- and post-bariatric patients, such as support groups and mental health professionals who specialize in this population, would also enhance not only dietary adherence but also patient emotional well-being after surgery.

The ethnographic research, however, suggests a more complex picture, where more sophisticated discussions within healthcare settings may be needed to move. In particular, clinicians perceive that low compliance leads to feelings of being stigmatized, whereas patients find the reverse relationship to be more accurate. This analysis of weight-related stigma within healthcare contexts reminds us that stigma is always created iteratively, and by both those feeling the stigma and those seen as creating it (Pescosolido et al., 2008; Pescosolido, 2013). As a result, we need robust mixed-method and longitudinal analyses to fully capture and then address properly how and why these types of stigmas persist. Thus, if providers—and patients who self-identify as adherent—frame negative encounters with certain patients in clinical contexts as an issue of adherence, not stigma, then promoting greater dialogue between providers and patients on the issue of “adherence” vs. “stigma” could be helpful in bringing the perspectives into closer alignment.

## Author contributions

The authors' responsibilities were as follows- AB, AW, and ST designed the research study and developed the protocols; ST conducted the qualitative interview research and managed survey data collection; SH, AB, and DR designed the quantitative analysis and SH analyzed the quantitative data; ST and AW designed and conducted the qualitative analysis. All authors wrote, read, and approved the final manuscript; DR had primary responsibility for final content.

## Funding

This project was supported in part by The Virginia G. Piper Charitable Trust through an award to Mayo Clinic/ASU Obesity Solutions.

### Conflict of interest statement

The authors declare that the research was conducted in the absence of any commercial or financial relationships that could be construed as a potential conflict of interest.
